# Impact of sink location on hand hygiene compliance after care of patients with *Clostridium difficile* infection: a cross-sectional study

**DOI:** 10.1186/s12879-016-1535-x

**Published:** 2016-05-16

**Authors:** Alexander Deyneko, Fernanda Cordeiro, Laurie Berlin, Debby Ben-David, Silvana Perna, Yves Longtin

**Affiliations:** Infection Prevention and Control Unit, Jewish General Hospital – SMBD, 3755 Côte-Sainte-Catherine Road, Room E-0057, Montreal, QC H3T 1E2 Canada; Chaim Sheba Medical Center, Tel Hashomer, Israel; McGill University Faculty of Medicine, Montreal, Canada

**Keywords:** Hand hygiene, *Clostridium difficile*, Handwashing, Additional precautions, Sinks, Epidemiology

## Abstract

**Background:**

The impact of sink location on hand washing compliance after contact with patients with *Clostridium difficile* infection (CDI) is poorly understood. The aim of this study is to determine the location of hand wash sinks available to healthcare workers (HCWs) after caring for patients with CDI and to assess the impact on hand washing compliance.

**Methods:**

We performed a cross-sectional study in a 637-bed tertiary care hospital, Canada. HCW hand hygiene compliance after contact with CDI patients was measured through direct unobtrusive observations. Location of sinks in relation with the patients’ rooms was assessed on the day of diagnosis. Predictors of compliance were assessed through univariate and multivariate logistic regression.

**Results:**

247 hand hygiene opportunities following care of a CDI patient were observed. Glove use compliance was 85.4 % (211/247), but hand washing compliance after care of CDI patients was only 14.2 % (35/247). Hand rubbing was performed instead of hand washing in 33.2 % of opportunities (82/247). The median distance between the patient zone of CDI patients and the nearest sink was 13.1 m (interquartile range, 7.6-23.2). Sinks were directly visible upon exiting the patient’s room on only 33.2 % (82/247) occasions. By multivariate analysis, an increasing distance between the patient zone and the nearest sink was inversely associated with hand washing compliance (adjusted OR, 0.90, 95 % CI, 0.84-0.97; *P* = 0.008), while proper timing of glove removal upon leaving the patient zone was directly associated with hand washing compliance (adjusted OR, 14.87; 95 % CI, 1.93-114.43; *P* = 0.01).

**Conclusions:**

Hand washing compliance following contact with patients with *C. difficile* infections was low. Poor access to sinks is associated with decreased hand washing compliance. Improvement strategies are urgently needed.

## Background

Hand hygiene is widely regarded as the most important infection control measure [1, 2]. There are 2 different ways to perform hand hygiene: hand washing with soap and water, or hand rubbing with an alcohol-based hand rub solution (ABHRS) [1, 2]. Over the past few decades, hand rubbing has gradually replaced hand washing and is now the preferred hand cleansing method in healthcare settings [2]. Its numerous advantages over soap and water include faster use, greater antimicrobial efficacy, and availability at the point of care [3]. However, one of the very few limitations of ABHRS resides in its lack of activity against spore-forming organisms, most notably *Clostridium difficile* [4–6]. For this reason, hand washing — not hand rubbing — is frequently recommended after caring for patients infected with *C. difficile* [2, 7].

The increasing popularity of ABHRS has undoubtedly led to a massive improvement in the quality of care. By contrast, hand washing as a preventive measure is now receiving less attention from researchers. For example, compliance with hand washing after caring for patients with *Clostridium difficile* infections (CDI), and factors influencing healthcare workers’ (HCW) compliance with hand washing in this context remain largely unknown, including the potential impact of easy access to hand wash sinks.

The primary goals of our study were to evaluate the location of sinks available for HCWs to hand wash after caring for CDI patients, and to investigate the relationship between sink location and HCWs’ compliance with hand washing.

## Methods

### Design and setting

This observational cross-sectional study was conducted on 15 wards of the Jewish General Hospital, Montreal, Canada, a 637-bed, tertiary medical centre. In 2013, 166 cases of healthcare-associated CDI occurred in our institution (incidence rate, 8.3/10,000 patient-days), due to a great extent to the high prevalence (55 %) of the epidemic NAP1/057 strain. Global hand hygiene compliance rate in 2013 was 47 % (1721/3635 opportunities). The hospital has 4 main types of accommodation for patient hospitalization: private rooms and 2-, 3- and 4-bedded rooms. Intensive care units (ICU) and maternity and psychiatry wards were excluded from the study as their configurations are very different from regular care units. On average, there was 1 sink per 10.7 beds available for HCWs on the study wards. The Jewish General Hospital Institutional Review Board approved the study as a quality improvement project.

### Survey of sinks

We determined the location of sinks available to HCWs to hand wash after contact with patients with CDI. Institutional policy requires HCWs to wear gloves and perform hand washing at the closest available sink after contact with these patients or their immediate surroundings (also called the “patient zone”) [2]. In accordance with national recommendations, the use of patient personal sinks (i.e., those present at the bedside for their personal hygiene) is not permitted as they are likely to be contaminated with spores [8]. We used the infection control electronic database to identify the rooms that accommodated ≥1 patient with CDI between April 6, 2012 and March 31, 2013. An auditor then visited the premises and identified the nearest available sink outside of the patient zone and recorded the following information: location (e.g., hallway, clean utility, nursing station, medication room, etc.); distance from the patient zone; whether the sink was directly visible from the patient’s room; and if it was not directly visible upon exiting the patient room, the number of 90° turns required to reach the sink. Distances were measured with a measuring wheel in meters. As newly diagnosed patients are usually transferred to a single patient room following CDI diagnosis, all these data were recorded twice for every patient: 1) on the day of diagnosis of CDI, and 2) 24 h after diagnosis. This allowed to determine whether patient transfer to a private room had any impact on the proximity of sinks.

### Handwashing compliance audits

From September to December 2013, an audit of HCWs’ hand hygiene compliance after CDI patient care was performed. This survey was conducted as part of our routine audits mandated by Accreditation Canada and performed by trained observers through direct unobtrusive observation according to the World Health Organization (WHO) recommendations [2]. Only opportunities after contact with CDI patients or surfaces within the patient zone (WHO, Moments 4 and 5; Canada, Moment 4) were audited [2]. Glove use, proper timing of glove removal (i.e., removal after leaving the patient zone and before touching any surface outside of the patient zone) and the type of hand cleansing method (rubbing or washing) were recorded. To decrease the Hawthorne effect, audits were limited to 30 min sessions and performed by a HCW who did not work in the areas where the audits were conducted [2].

### Analysis

Standard descriptive statistics were used to calculate the proportion of room types, location of sinks, median distances, and the number of 90° turns between patient rooms and sinks. Medians and interquartile range (IQR) were used for non-normally distributed data. Geospatial characteristics at *t* = 0 h and *t* = 24 h were compared. Medians were compared using the Mann–Whitney test for unpaired variables and the Wilcoxon rank-sum test for paired samples. Categorical data were compared using *χ*^2^ and Fisher’s exact test.

Predictors of hand washing compliance were analyzed by univariate and multivariate logistic regression. To adjust for potential confounders, all variables found to be associated with hand washing (*P* < 0.05) by univariate analysis were considered for inclusion in a multivariate model [9]. Five potential variables were identified: “glove use”, “direct visualisation of the sink”, “number of 90° turns”, “proper glove removal” and “distance to the sink”. The variable “direct visualization of the sink” was removed as it was strongly correlated with the variable “number of 90° turns”. All data related to a single respondent were excluded when any of the variables included in the model had missing values. Given that HCWs who did not wear gloves during patient care had a missing value for the variable “proper glove removal’, the variable “glove use” would have been uniformly positive in a multivariate model; it was thus excluded from the final model. Hence, we built a forced-entry model with the variables “proper glove removal”, “distance to sink”, and “number of 90° turns”. The magnitude of the association between outcomes and explanatory variables was measured by odds ratios (OR) and corresponding 95 % confidence intervals (CI). The number of 90° turns was treated as an ordinal variable for the univariate analysis to facilitate reporting, but as a continuous variable for the multivariate analysis. All tests were two-tailed and a *P*-value <0.05 was defined as statistically significant. Statistical analyses were performed with R (version 3.02; R Foundation for Statistical Computing; Vienna, Austria) and PASW Statistics version 18.0 (SPSS Inc. Chicago, IL).

## Results

### Sink survey

#### Patient accommodation

In total, the location of 183 patients with CDI in relation to sinks were analyzed. On the day of diagnosis, most patients (114 patients; 62.3 %) were located in a 2-bedded room, followed by a 3- or 4-bedded room (44 patients; 24.0 %; Table 1), whereas only 25 patients (13.7 %) were located in private rooms. Twenty-four hours after diagnosis, the proportion of patients housed in private rooms had increased significantly to 45.2 % (*P* < 0.001), whereas the proportion housed in 2-bedded or 3/4-bedded rooms had decreased significantly to 50.6 % and 4.2 %, respectively (*P* ≤ 0.04). In addition, 13 patients had also been discharged and 2 patients had been moved to a unit not included in the study.

**Table 1:** Characteristics of rooms hosting patients with *Clostridium difficile* infections, Jewish General Hospital, Canada, April 2012- March 2013

	On the day of diagnosis	24 h after diagnosis	*P*-value
	*N* = 183	*n* = 168^a^	
Room type (%)			
Private	25 (13.7 %)	76 (45.2 %)	<0.001
2-bedded room	114 (62.3 %)	85 (50.6 %)	0.04
3-4-bedded room	44 (24.0 %)	7 (4.2 %)	<0.001
Location of the closest available sink for HCW handwash (%)			
Nursing station	92 (50.3 %)	79 (47.0 %)	0.61
Hallway	78 (42.6 %)	83 (49.4 %)	0.24
Medication room	9 (4.9 %)	4 (2.4 %)	0.26
Clean utility room	2 (1.1 %)	2 (1.2 %)	1.00
Other	2 (1.1 %)	0 (0 %)	0.49
Median distance between patient room and closest sink (meters) (IQR)	11.6 (6.7–18.9)	12.5 (6.7–23.2)	0.005
Private room	9.8 (4.0–22)	16.2 (6.4–25.6)	0.21
2 bedded room	10.5 (6.3–18.9)	10.4 (7.0–20.1)	0.60
3-4 bedded room	12.8 (8.2–20.7)	18.9 (13.4–23.2)	0.22
Path from room to nearest sink (%)			
Direct line from patient room	60 (32.8 %)	62 (36.9 %)	0.49
One 90° turn required to reach sink	30 (16.4 %)	22 (13.1 %)	0.47
Two or more 90° turns required to reach sink	93 (50.8 %)	84 (50.0 %)	0.96

^a^24 h after diagnosis, 15 patients were excluded as they had been discharged (*n* = 13) or transferred to a non-study unit (*n* = 2)

Abbreviation: *IQR* interquartile range

### Location of sinks in relation to CDI patients

On the day of CDI diagnosis, half of all the closest sinks (92/183 sinks; 50.3 %) were located in the nursing station. Hallways were the second most common location (478/183; 42.6 %), followed by sinks located in medication rooms (9/183; 4.9 %) and other locations (4/183; 2.2 %). The location of the nearest sinks on the day of diagnosis and 24 h later were comparable (*P* > 0.05). Globally, the median distance between the patient’s room and the nearest sink on the day of diagnosis was 11.6 m (IQR, 6.7–18.9), with a minimum of 1.2 m and a maximum of 37.8 m. Twenty-four hours after diagnosis, this distance had increased slightly but significantly to 12.5 m (IQR, 6.7–23.2) (*P* = 0.005 by Wilcoxon rank-sum test). On both the day of diagnosis and 24 h after diagnosis, only one-third of sinks were directly visible upon exiting the patient zone. In half of all occasions, HCWs needed to perform 2 or more 90° turns to reach the nearest sink.

### Hand hygiene compliance after care of CDI patients

In total, 247 hand hygiene opportunities following care of a CDI patient were observed (Table 2). Hand washing occurred in 35/247 (14.2 %) of opportunities. In two-thirds of these cases (24/35; 68.6 %), hand washing occurred in isolation, whereas it occurred in combination with hand rubbing in the remainder. Hand rubbing without hand washing was performed in one-third of opportunities (82/247; 33.2 %). Gloves were used most of the time (211; 85.4 %); proper timing of removal occurred in 160/211 (75.8 %).

**Table 2:** Factors associated with performance of hand washing after exposure to *Clostridium difficile*, Jewish General Hospital, Canada, April 2012- March 2013

Characteristics	Hand washing performed (%)	Hand washing not perfomed (%)	*P*-value
Type of healthcare worker			
Nurse	18/94 (19.1 %)	76/94 (80.9 %)	ref
MD	2/12 (16.7 %)	10/12 (83.3 %)	0.90
Medical resident/student	6/32 (18.8 %)	26/32 (81.3 %)	0.91
Orderly	3/36 (8.3 %)	33/36 (91.7 %)	0.95
Meal service	0/40 (0 %)	40/40 (100 %)	0.24
Other^a^	6/33 (18.2 %)	27/33 (81.8 %)	0.99
Hand hygiene			
Hand rubbing alone	0/82 (0 %)	82/82 (100 %)	n/a
Hand washing alone	24/24 (100 %)	0/24 (0 %)	n/a
Hand rubbing followed by hand washing	11/11 (100 %)	0/11 (0 %)	n/a
None performed	0/130 (0 %)	130/130 (100 %)	n/a
Glove use			
Glove use while caring for patient	35/211 (16.6 %)	176/211 (83.4 %)	ref
No glove use while caring for patients	0/36 (0 %)	36/36 (100 %)	0.02
Glove removal^b^			
Proper timing of glove removal after leaving patient zone^b^	35/160 (21.9 %)	125/160 (78.1 %)	Ref
Improper timing of glove removal after leaving patient zone^b^	0/51 (0 %)	51/51 (100 %)	<0.001
Location of nearest available sink			
Hallway	19/70 (27.1 %)	51/70 (72.9 %)	Ref
Nursing station	9/129 (7.0 %)	120/129 (93.0 %)	0.23
Medication room	4/28 (14.3 %)	24/28 (85.7 %)	0.27
Other	3/20 (15.0 %)	17/20 (85.0 %)	0.95
Median distance to sink (IQR)	7.6 (4.3–12.5)	14.9 (10.4–23.4)	<0.001
Number of turns required to reach sink			
None	21/82 (25.6 %)	61/82 (74.4 %)	Ref
One 90° turn	8/49 (16.3 %)	41/49 (83.7 %)	0.22
Two 90° turns	6/71 (8.5 %)	65/71 (91.5 %)	0.008
≥ Three 90^o^ turns	0/45 (0 %)	45/45 (100 %)	0.006
Visualization of sink from patient zone			
Sink within direct sight from patient room	21/82 (25.6 %)	61/82 (74.4 %)	Ref
Sink not in direct sight from patient room	14/165 (8.5 %)	151/165 (91.5 %)	<0.001

Abbreviations: *IQR* interquartile range, *n/a* not applicable, *Ref* reference

^a^Includes occupational therapist, physiotherapist, radiology technician, pharmacist

^b^Only among 211 healthcare workers who wore gloves while in the room

### Predictors of handwashing compliance

By univariate analysis, there was no association between the profession of the HCW and hand washing compliance (*P* > 0.05). The use of gloves was associated with a higher likelihood of hand washing: 16 % of HCWs who wore gloves while in the patient zone performed hand washing upon exiting, compared with none of those who did not wear gloves (*P* = 0.02). Among the 211 HCWs who wore gloves during patient care, proper timing of glove removal was associated with a greater compliance with hand washing compared with those who did not remove the gloves properly (21.9 % vs. 0 %; *P* < 0.001). The distance to the sink was strongly associated with hand washing compliance. The median distance to the nearest sink was only 7.6 m when it was correctly performed, whereas it was 14.9 m when it was omitted (*P* < 0.001). There was also a strong association between the number of 90° turns required to reach the sink and hand washing compliance. Compliance was highest when the sink was in a direct line from the patient zone (25.6 %), but steadily decreased to 16.3, 8.5 and 0 % when 1, 2 or ≥ 3 turns, respectively, were needed to reach the sink (*P* < 0.001 for trend). Finally, direct visualization of the sink from the patient zone was associated with a higher compliance than when it was not visible (25.6 % vs. 8.5 %, respectively; *P* < 0.001).

### Multivariate analysis

In a multivariate model, the distance to the sink was independently and inversely associated with hand washing (adjusted OR, 0.90; 95 % CI, 0.84–0.97; *P* = 0.008; Table 3). In addition, proper timing of glove removal was also associated with hand washing compliance (adjusted OR, 14.87; 95 % CI, 1.93–114.43; *P* = 0.01). Although predictors by univariate analysis, the number of 90° turns was not associated with hand washing in the multivariate analysis (*P* > 0.05).

**Table 3:** Factors associated with hand washing following *Clostridium difficile* exposure by multivariate analysis, Jewish General Hospital, Canada, April 2012- March 2013

Characteristic	Adjusted OR	95 % CI	*P*
Proper timing of glove removal upon exiting patient zone	14.87	1.93–114.43	0.01
Distance between patient zone and sink	0.90	0.84–0.97	0.008
Number of 90° turns to reach the sink	0.78	0.44–1.37	0.78

Analysis performed on 211 hand hygiene observations. Only observations during which healthcare workers wore gloves were included in the analysis. 36 observations (15 %) were excluded from the analysis due to missing data

Abbreviations: *OR* odds ratio, *CI* confidence interval

## Discussion

Understanding barriers and enablers that may influence compliance with hand washing is important to improve HCWs’ adherence to this life-saving procedure [2]. To the best of our knowledge, this study is the first to investigate factors associated with hand washing compliance specifically after contact with CDI patients. We demonstrated that distances between CDI patient rooms and hand washing sinks were sizeable and this well reflects the global shortage of sinks in our institution. This situation is not surprising considering that our hospital was built in 1934, a period when the importance of hand hygiene was under recognized.

CDI has become the most common healthcare-associated infection in the United States, affecting half a million patients every year and causing 29,000 deaths [10]. There is an urgent need to improve infection control measures to control this threat. According to international recommendations, newly diagnosed CDI patients should be rapidly transferred to a private room. However, this recommendation comes at a cost in our institution: 24 h after diagnosis, patients are located in a room that is even farther from a sink than on the day of diagnosis. This may be explained by the fact that private rooms tend to be located at the periphery of the units. Since half of the sinks in our study are located in the nursing station, transferring patients to a private room often moved them away from sinks. The need to balance the requirement for private room accommodation against a general lack of access to sinks near these private rooms illustrates perfectly the dilemmas routinely encountered by infection control teams. The situation is made even more complex by the fact that, contrary to ABHRS dispensers, adding extra sinks is complex and costly in aging buildings.

Glove use and proper glove removal was relatively high in our study, but hand washing compliance was low. Hand washing was performed only once every 7 opportunities, whereas hand rubbing was performed approximately one-third of the time. A study conducted in US hospitals before the implementation of ABHR dispensers measured very low hand washing compliance [11]. Our study suggests that similar conclusions could be drawn for hand washing compliance after contact with CDI patients.

Ensuring sufficient availability of ABHRS at the point of care is known to be critical to good hand hygiene compliance [2]. Our study shows that availability of sinks is also an independent predictor of hand washing compliance and demonstrates that an increased distance between the patient zone and the sink is associated with decreased hand washing compliance. Every additional meter that must be walked by the HCW to reach a sink decreased the likelihood of hand washing by approximately 10 %. These results are in contrast with those of another study that did not measure any decrease in hand washing compliance with decreasing sink-to-bed ratio [11].

A recent study investigated the relationship between sink visibility and hand wash basin use on paediatric ICUs and demonstrated that more visible sinks are used more often [12]. In our study, a similar indicator of sink visibility (number of 90° turns) was associated also with hand washing compliance by univariate analysis, but the association disappeared when taking other variables into account in a multivariate analysis.

We identified proper timing of glove removal as an independent predictor of compliance. HCWs who wore gloves and removed them properly when leaving the patient zone were 14 times more likely to hand wash as indicated. This finding most likely reflects the behaviour of HCWs who are more attune to good hand hygiene practices and we hypothesize that proper glove removal is a global marker of good infection control practices.

Our study has some limitations. The sample size was limited and the study was performed in a single institution with a limited number of observations. Still, we believe our study is valuable as it is, to our knowledge, the first to investigate predictors of hand washing compliance after care of patients with CDI. It is challenging to conduct hand hygiene audits focusing only on CDI patients as this patient population is relatively small and that long periods may elapse between opportunities. The number of observations limited the number of variables that could be included in the multivariate analysis [9]. There are few published data against which to compare our results and our findings will need to be confirmed by further studies [11]. The mechanisms through which sink location may impact compliance (for example, by improving workflow or by acting as a cue to action) could not be evaluated. Our study is not designed to determine whether increasing sink availability would improve in hand washing compliance. Studies on this question have provided conflicting data [13–15]. Our study is not designed to correlate hand hygiene compliance and CDI rates. In addition, it should be recalled that the choice of hand hygiene technique following the care of CDI patients is currently a matter of debate because of the paucity of clinical evidence to support it. Even though the WHO recommends hand washing instead of hand rubbing after care of CDI patients, [2] other organizations, such as the US Society of Healthcare Epidemiology of America (SHEA), allow hand rubbing after the care of most CDI patients and only recommend hand washing in the context of outbreaks or increased CDI rates [1, 16]. Following such a recommendation would have improved our measured compliance rate by more than 2-fold to 47.4 %. However, with its very high CDI rates, our institution was considered hyperendemic and fulfils the SHEA criteria for hand washing in this context. A recent study has shown also that up to 24 % of HCWs’ hands are contaminated with *C. difficile* spores after caring for a CDI patient, despite glove use [17]. This finding supports the need to hand wash after glove removal, rather than hand rub.

## Conclusions

In conclusion, there is an association between the location of hand wash sinks and HCW’s compliance with hand washing after caring for patients with CDI. There is a need to identify mitigating strategies to continue to improve patient safety. The worldwide re-emergence of CDI should trigger a renewed interest in research on hand washing.

### Ethics approval

The Jewish General Hospital Institutional Review Board approved the study as a quality improvement project. No consent to participate was required.

## Availability of data and material

Not applicable.

## Abbreviations

ABHRS: alcohol-based handrub solution
CDI: *Clostridium difficile* infection
HCW: healthcare worker
ICU: Intensive care unit
IQR: interquartile range
OR: odds ratio
WHO: World Health Organization
